# Glutamate signals through mGluR2 to control Schwann cell differentiation and proliferation

**DOI:** 10.1038/srep29856

**Published:** 2016-07-19

**Authors:** Fuminori Saitoh, Shuji Wakatsuki, Shinji Tokunaga, Hiroki Fujieda, Toshiyuki Araki

**Affiliations:** 1 Department of Peripheral Nervous System Research National Institute of Neuroscience, National Center of Neurology and Psychiatry, 4-1-1 Ogawa-higashi, Kodaira, Tokyo 187-8502, Japan; 2 Department of Anatomy, School of Medicine, Tokyo Women’s Medical University, Tokyo 162-8666, Japan

## Abstract

Rapid saltatory nerve conduction is facilitated by myelin structure, which is produced by Schwann cells (SC) in the peripheral nervous system (PNS). Proper development and degeneration/regeneration after injury requires regulated phenotypic changes of SC. We have previously shown that glutamate can induce SC proliferation in culture. Here we show that glutamate signals through metabotropic glutamate receptor 2 (mGluR2) to induce Erk phosphorylation in SC. mGluR2-elicited Erk phosphorylation requires ErbB2/3 receptor tyrosine kinase phosphorylation to limit the signaling cascade that promotes phosphorylation of Erk, but not Akt. We found that Gβγ and Src are involved in subcellular signaling downstream of mGluR2. We also found that glutamate can transform myelinating SC to proliferating SC, while inhibition of mGluR2 signaling can inhibit demyelination of injured nerves *in vivo*. These data suggest pathophysiological significance of mGluR2 signaling in PNS and its possible therapeutic importance to combat demyelinating disorders including Charcot-Marie-Tooth disease.

Rapid saltatory conduction of electrical signals along the distance of an axon is facilitated by myelin, a lipid-rich ionic insulator that wraps around the axon^1^,^2^. In the peripheral nervous system (PNS), myelination is accomplished by the peripheral type of glial cells, i.e., Schwann cells (SC). In PNS development, axons regulate SC differentiation into myelinating and non-myelinating cell populations. Once SC detach from axons by disease or damage, SC rapidly and drastically change their phenotype to become de-differentiated, which is crucial to facilitate axonal regeneration^3^.

Signaling that translates humoral/contact-mediated stimulation to SC differentiation has been thoroughly examined to better understand the mechanism of myelination in the PNS. Neuregulin-1 (Nrg1)-ErbB2/3 signal is known to play a major role in SC differentiation and proliferation. SC precursors that associate with peripheral axons express the tyrosine kinase receptors ErbB2/3^4^, as well as, the neuronally produced Nrg1, for migration, proliferation, and differentiation^5^,^6^. Nrg1 can also be produced by adult SC in degenerating nerves to promote the de-differentiated SC phenotype^7^. ErbB2 activation in SC elicits multiple intracellular signaling pathways, including ones that activate Erk and Akt^8^,^9^,^10^. cAMP is also known as a key regulatory factor for SC proliferation and differentiation. cAMP-stimulating agents increase the potency of Nrg1; cAMP can also upregulate the expression of a number of myelin genes such as: myelin protein zero (P0) and the transcription factor SCIP/Oct-6/Tst-1, which are essential regulators of SC differentiation into a myelinating phenotype^11^. The best known downstream effector of cAMP is protein kinase A (PKA). Inhibition of PKA has been shown to block the morphological changes in SC, which is induced by raising cAMP levels^12^.

It has become increasingly clear that the undifferentiated/differentiated phenotypes of SC are tightly regulated by a cascade of intracellular signaling events, and an orchestrated series of gene expression pathways of regulatory proteins and molecules found on the cell surface and extracellular matrix^13^,^14^. For instance, the de-differentiation signals for SC invariably overlap with proliferation, particularly in *in vivo* situations. Nrg1 may be the most well-characterized and only example of such molecule that can elicit multiple SC intracellular signaling pathways, but the knowledge with regard to the molecular mechanism to coordinate these multiple events in SC is still limited.

We previously reported that expression of glutamine synthetase (GS) in SC promotes their myelination phenotype. GS is an enzyme that catalyzes conversion of glutamate into glutamine. We previously showed by using *in vitro* myelination experiments that increased glutamate concentration inhibited myelination and yet promoted SC proliferation by activating metabotropic glutamate receptor (mGluR) signaling. GS expression decreases glutamate concentration, and thereby promotes myelination^15^. In this report, we show that mGluR activation plays a role in promoting SC proliferation and de-differentiation by eliciting intracellular signaling downstream of a G-protein coupled receptor, while mGluR inhibition promotes myelination. Interestingly, mGluR activation enhances Nrg1-induced Erk phosphorylation that promotes proliferation and de-differentiation of SC, but not Akt phosphorylation that results in SC migration and/or sorting. mGluR signaling showed significant effect on modulating SC phenotype *in vivo*. Our data suggest that controlling glutamate levels in peripheral nerve is crucial for SC proliferation, as well as differentiation.

## Results

### Glutamate enhances Erk phosphorylation via mGluR2 on SC

**Figure 1:**
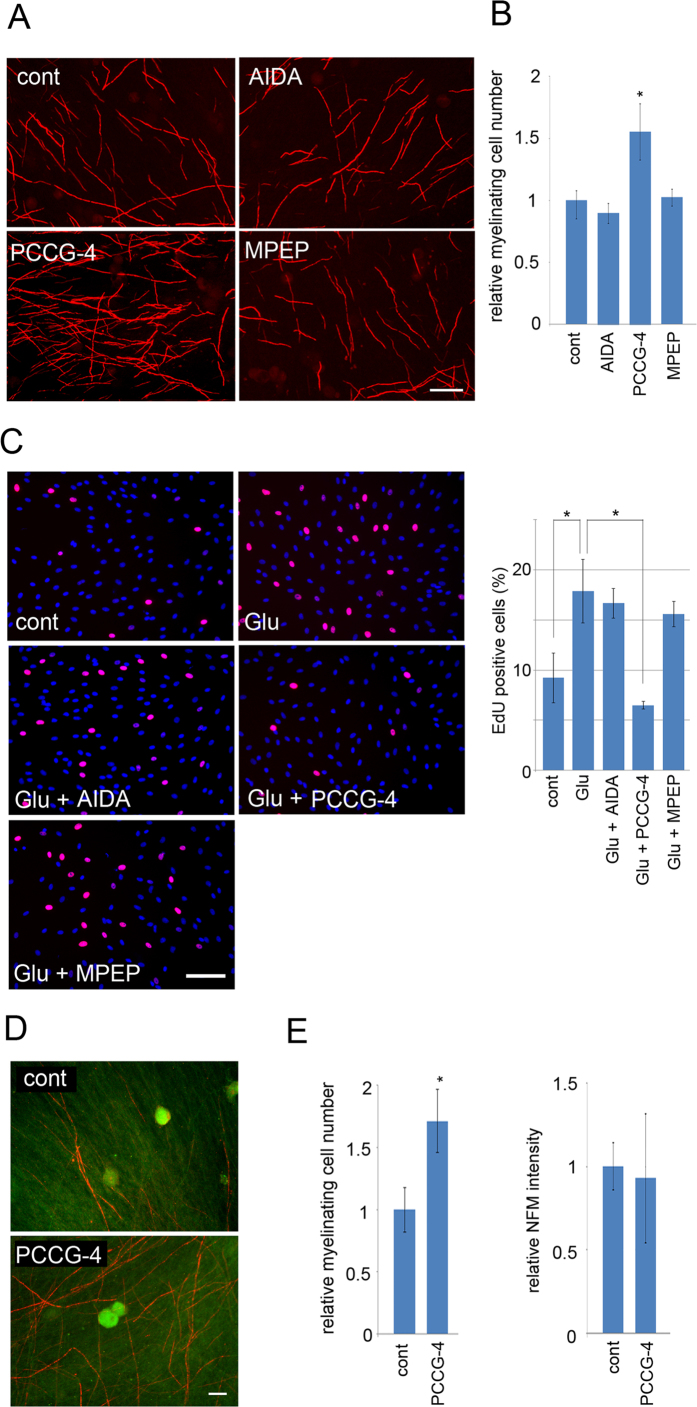
Inhibiting mGluR2/3 signaling promotes myelination. (**A,B**) *In vitro* myelination was performed using a co-culture of DRG neurons and SC with indicated mGluR antagonists (AIDA for mGluR1, PCCG-4 for mGluR2/3, and MPEP for mGluR5). Resultant myelination profiles were visualized by MBP immunocytochemistry. Representative myelination profiles (**A**) and their quantification data (**B**) are shown. Scale bar = 100 μm. For quantification, myelination profiles were counted from five randomly selected fields under a microscope using a 10x objective lens. The number of profiles relative to no inhibitor control is shown (mean profile numbers of 5 independent experiments ± SD). Asterisks indicate significant difference from control (Student’s t test, *p < 0.05). Note that myelination was promoted by PCCG-4, an mGluR2/3 antagonist. (**C**) Representative photomicrographs and quantification of EdU-positive SC in the proliferation assay with indicated conditions. Cells were co-stained with DAPI. Scale bar = 250 μm. For quantification, the percentage of EdU-possitive cells to the total number of cells were calculated in each condition (n = 3, mean ± SD). Statistical analysis was performed by Student’s t-test. Asterisks indicate significant difference (*p < 0.05). (**D,E**) Representative photomicrographs and quantification of neurofilament M (NFM) and MBP immunoreactivity of the DRG neuron-SC co-culture. Scale bar = 50 μm. For quantification in (**E**) the numbers of myelination profiles visualized by MBP staining and the intensity of NFM immunoreactivity were analyzed from five randomly selected fields under a microscope using a 20x objective lens (mean ± SD, n = 3). Asterisks indicate significant difference from control (Student’s t test, *p < 0.05).

In our previous report, we showed that forced expression of GS resulted in increased myelination *in vitro*, and that glutamate along with an mGluR agonist promotes SC proliferation. These data suggested that glutamate affects the differentiation status of SC via mGluR on SC. To show that the myelination-promoting effect of GS expression in SC is mediated by mGluR signaling, we first examined the effects of mGluR inhibitors on SC myelination and glutamate-induced SC proliferation *in vitro*. We found that application of PCCG-4, an mGluR2/3 antagonist, significantly promoted myelination to an extent similar to GS overexpression in SC (Fig. 1). We also found that PCCG-4 inhibited glutamate-induced SC proliferation. These data suggest that glutamate affects SC proliferation and differentiation by signaling via mGluR2/3.

**Figure 2:**
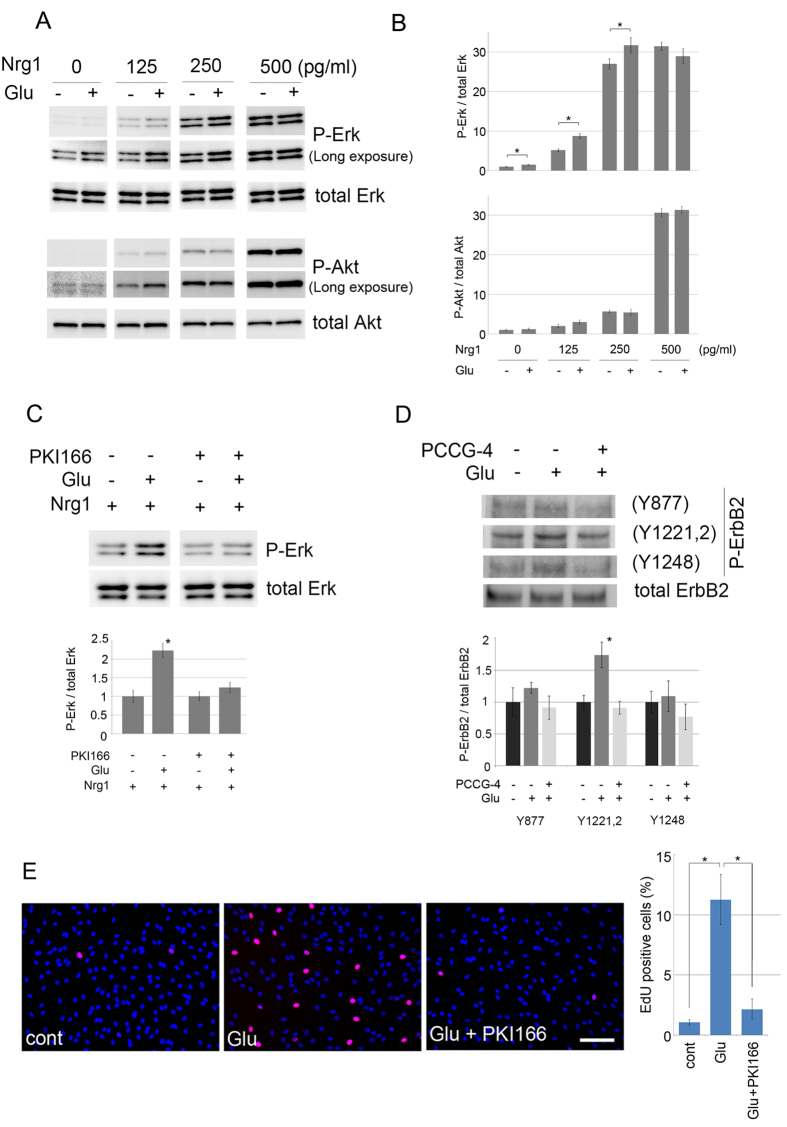
ErbB2 phosphorylation in response to glutamate in SC causes increased phosphorylation of Erk but not Akt. (**A,B**) Representative immunoblot images (**A**) and quantified expression levels (**B**) of phosphorylated/total Erk and Akt in primary cultured SC after treatment with glutamate and Nrg1 at varying concentrations as indicated for 30 min. The quantified expression level was normalized to the total Erk or Akt expression level, relative to the condition without glutamate/Nrg1 (mean ± SD, n = 3). The asterisks indicate significant difference between glutamate +/− conditions at each Nrg1 concentration (Student’s t test, *P < 0.05) (**C**) Representative immunoblot images (top) and quantified expression levels (bottom) of phosphorylated/total Erk in primary cultured SC in response to glutamate in the presence or absence of PKI166. The quantified expression level was normalized to the total Erk expression level, relative to the condition without glutamate (mean ± SD, n = 3). Note that glutamate-induced increase of Erk phosphorylation is strongly inhibited by PKI166. The asterisk indicates significant difference (Student’s t test, *P < 0.05) (**D**) Representative immunoblot images (top) and quantified expression levels (bottom) of phosphorylated ErbB2 at indicated tyrosine residues in the presence of glutamate with/without mGluR2,3 antagonist application. The quantified expression level was normalized to the total ErbB2 expression level, relative to the condition without glutamate (mean ± SD, n = 3). Total ErbB2 expression levels serve as a loading control. Note that glutamate-induced ErbB2 phosphorylation is inhibited by PCCG-4. (**E**) Representative photomicrographs and quantification of EdU-positive cells in the proliferation assay. SC were treated with 2 mM glutamate, together with PKI166 (200 nM). The proliferating cells were labeled 22 h later by adding EdU (20 μM) for another 2 h. Cells were co-stained with DAPI. Scale bar = 250 μm. The percentage of EdU-possitive cells to the total number of cells were calculated in each condition (n = 3, Mean ± SD). Statistical analysis was performed by Student’s t-test, Asterisks indicate significant difference (p < 0.05).

Previous reports indicated that subcellular signaling resulting in proliferation/de-differentiation of SC is mostly associated with Ras-Raf-Erk signaling and/or Akt^8^. Other reports have shown that Erk signaling in SC is associated with demyelination and dedifferentiation^9^. Whereas, Akt activation in SC is elicited by extracellular matrix and/or cell-to-cell contact, and is involved in their migration and radial sorting^7^,^10^. Therefore, to dissect mGluR2/3-elicited signaling in SC, we first examined the phosphorylation status of Erk and Akt after application of glutamate to primary cultured SC. We found that glutamate induces a weak but significant increase of Erk phosphorylation, and does not affect phosphorylation status of Akt in SC (Fig. 2A,B). We also found that glutamate accelerates Nrg1-induced Erk phosphorylation, but does not affect Nrg1-induced Akt phosphorylation (Fig. 2A,B). These results suggest that glutamate may elicit SC signaling by modulating ErbB2 receptor-mediated subcellular signaling. To determine if ErbB2 is required for glutamate-induced SC signaling, we examined Erk phosphorylation in response to glutamate under the presence of PKI166, a potent inhibitor of kinase activity of ErbB2 and EGFR. We found that PKI166 shuts down Erk phosphorylation in response to Nrg1 and/or glutamate (Fig. 2C). We also found that PKI166 inhibited the glutamate-induced SC proliferation in culture (Fig. 2E). These results suggest that mGluR2/3 signaling in SC requires ErbB2 receptor to induce Erk phosphorylation and resultant SC proliferation. To further characterize the mechanism of inducing Erk-specific phosphorylation downstream of ErbB2 receptor signaling, we employed antibodies against specific phosphorylated tyrosine residues of ErbB2 to determine which residues are phosphorylated in response to glutamate application on SC. Among possible phosphorylation sites, we found that glutamate strongly induces phosphorylation of Y1221, more so than other tyrosine residues including Y877 or Y1248 (Fig. 2D). Y1221/1222 phosphorylation is known to constitute a docking site for cytoplasmic proteins of the ErbB2 signal transduction cascade^4^,^16^,^17^. Taken together, these results suggest that glutamate elicits a subset of subcellular signaling downstream of ErbB2 receptor activation by inducing ErbB2 phosphorylation in a manner independent from ligand binding.

**Figure 3:**
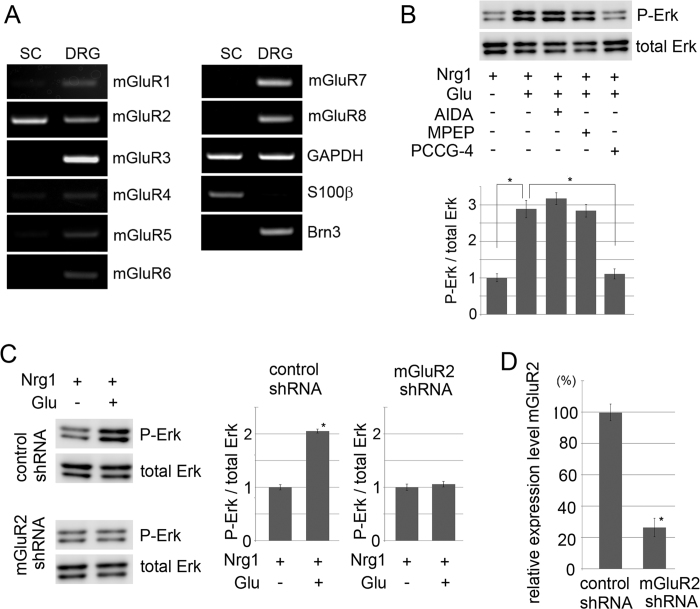
Glutamate induces Erk phosphorylation in SC via mGluR2. (**A**) Representative images of RTPCR analysis for expression of mGluR family members in cultured SC and DRG neurons. The expressions of GAPDH, S100β (a Schwann cell marker) and Brn3a (a neuron marker) mRNA were also analyzed for control. (**B**) Representative images of immunoblot analysis (top) and quantified expression levels (bottom) of phosphorylated Erk levels in SC with glutamate stimulation as indicated. The quantified expression level was normalized to the total Erk expression level, relative to the condition without glutamate (n = 3, Mean ± SD). Note that glutamate-induced Erk phosphorylation is cancelled by PCCG-4, but not by AIDA or MPEP. The asterisks indicate significant difference (Student’s t test, *P < 0.05). (**C,D)** Representative images of immunoblot analysis (left) and quantified expression levels (right) of phosphorylated Erk expression in SC with RNAi-mediated down-regulation of mGluR2. The quantified expression level was normalized to the total Erk expression level, relative to the condition without glutamate (n = 3, Mean ± SD). Efficiency of RNAi-mediated down-regulation of mGluR2 expression performed in (**C**) is shown in (**D**) (n = 3, mean expression levels relative to control ± SD). The asterisks indicate significant difference (Student’s t test, *P < 0.05).

**Figure 4:**
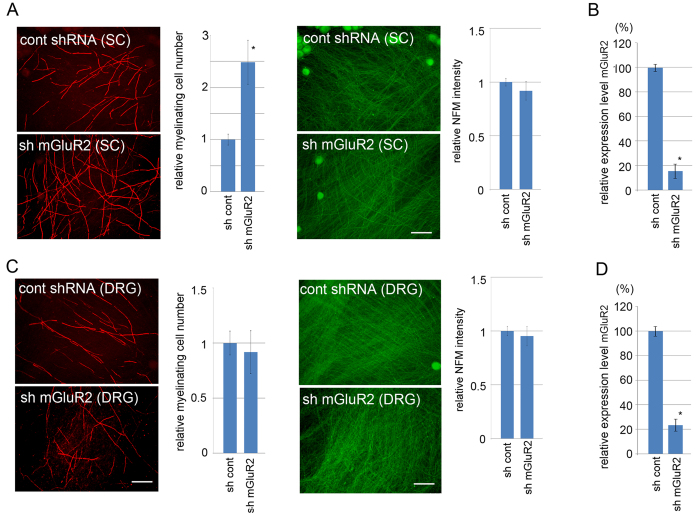
Down-regulation of mGluR2 in SC promotes myelination. *In vitro* myelination was performed using a co-culture of DRG neurons and Schwann cells. SC expressing mGluR2 shRNA were used in (**A**) and DRG neuros expressing mGluR2 shRNA were used in (**C**). Representative photomicrographs showing myelination profiles by MBP immunoreactivity and their quantification data are shown. Scale bars = 100 μm. For quantification, the number of myelinating profiles relative to the number obtained from control shRNA-expressing condition is calculated from 5 independent experiments for each condition (mean ± SD). Representative photomicrographs and quantification of NFM staining of the co-cultures are also shown to demonstrate that the down-regulation of mGluR2 does not affect neurite density in the co-culture. Evaluation of the efficiency of RNAi-mediated down-regulation of mGluR2 expression performed in (**A,C**) is demonstrated by qRTPCR in (**B,D**) respectively. (n = 3, mean expression levels relative to control ± SD). Note that myelination was promoted by down-regulation of mGluR2 in SC but not in DRG neurons. The asterisks indicate significant difference (Student’s t test, *P < 0.05).

We showed in Fig. 1 that application of an mGluR2/3-specific inhibitor promoted myelination *in vitro*. To characterize glutamate-elicited intracellular signaling in SC, we tried to determine the receptor subtype(s) responsible for signaling. For this purpose, we examined mRNA expression of mGluR family members by RT-PCR. We found that mGluR2 showed detectable mRNA expression in primary cultured SC (Fig. 3A). Together with the data in Fig. 1, our data suggest that glutamate signals through mGluR2 on SC to affect SC proliferation and differentiation. To confirm the involvement of mGluR2 in the glutamate-induced effect on SC, we perfomed shRNA-mediated mGluR2 down-regulation in SC or DRG neurons in *in vitro* myelination experiments (Fig. 4) We found that mGluR2 down-regulation in SC resulted in increased myelination in culture as observed by using a mGluR inhibitor. These results indicate that mGluR2 on SC is the signaling gate for glutamate to affect SC proliferation and differentiation.

To analyze mGluR2-mediated signaling in SC, we first confirmed that mGluR2 activation is necessary for glutamate-induced Erk phosphorylation. For this purpose we employed different mGluR antagonists and applied them with glutamate and/or Nrg1 on primary cultured SC. Among different mGluR antagonists, we found that glutamate-induced Erk phosphorylation was suppressed by PCCG-4, but not by AIDA (an mGluR1 antagonist) or MPEP (an mGluR5 antagonist) (Fig. 3B). These data suggest that glutamate induces SC proliferation by way of mGluR2. To further confirm that mGluR2 is the signaling gate for glutamate on SC, we down-regulated expression of mGluR2 by RNAi and examined its effect on phosphorylated Erk levels. We observed that SC infected with lentiviral expression vector for mGluR2 shRNA abolished glutamate-mediated Erk phosphorylation (Fig. 3C,D). We also found that mGluR2 shRNA inhibited the glutamate-induced SC proliferation in culture (data not shown). These results indicate that glutamate enhances Erk activation, and thereby induces proliferation via mGluR2.

### mGluR elicits intracellular signaling involving Gαi and Gβγ in SC

**Figure 5:**
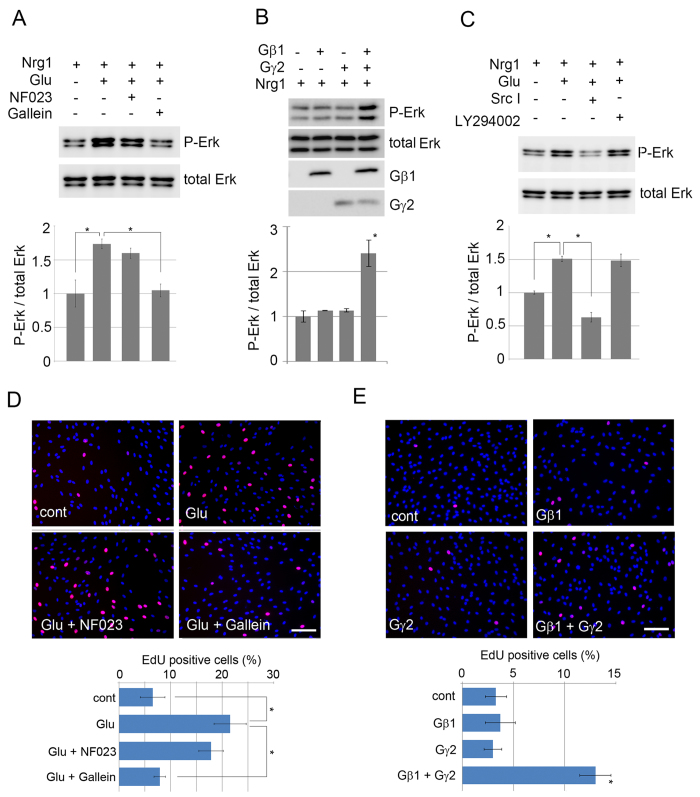
Gβγ and Src serve as signaling components downstream of mGluR2. (**A**) Representative images of immunoblot analysis (top) and quantified expression levels (bottom) of glutamate-induced potentiation of phosphorylated Erk expression in primary cultured SC in the presence or absence of NF023 (an inhibitor for Gαi) or gallein (an inhibitor for Gβγ). The quantified phosphorylated Erk expression level was normalized to the total Erk level, relative to the condition without glutamate (n = 3, Mean ± SD). The asterisks indicate significant difference (Student’s t test, *P < 0.05). Note that gallein inhibited glutamate-induced Erk phosphorylation, while NF023 showed no effect. (**B**) Representative images of immunoblot analysis(top) and quantified expression levels (bottom) of phosphorylated Erk expression in primary cultured SC overexpressing Gβ1 and/or Gγ2 proteins with/without Nrg1 stimulation. The quantified expression level was normalized to the total Erk level, relative to the condition without Gβ1 or Gγ2 (n = 3, Mean ± SD). The asterisks indicate significant difference (Student’s t test, *P < 0.05). Note that combined expression of Gβ1 and Gγ2 increases Erk phosphorylation. (**C**) Representative images of immunoblot analysis (top) and quantified expression levels (bottom) of phosphorylated Erk expression in primary cultured SC stimulated by indicated reagents. The quantified expression level was normalized to the total Erk expression level, relative to the condition without glutamate (n = 3, Mean ± SD). The asterisks indicate significant difference (Student’s t test, *P < 0.05). Note that glutamate-induced Erk phosphorylation is significantly inhibited by Src I, but not LY294002. (**D,E**) Representative photomicrographs and quantification of EdU-positive cells in the proliferation assay. Schwann cells were treated with 2 mM glutamate, together with NF023 (10 μM) or gallein (400 nM) in (**D**), or transfected with His-Gβ1 and/or His-Gγ2 expression vectors using electroporator in (**E**). The proliferating cells were labeled 22 h later by adding EdU (20 μM) for another 2 h. Cells were co-stained with DAPI. Scale bar = 250 μm. The percentage of EdU-possitive cells to the total number of cells were calculated in each condition (n = 3, Mean ± SD). Statistical analysis was performed by Student’s t-test. The asterisk indicates significant difference (p < 0.05).

mGluR2 is known to elicit intracellular signaling by association with Gi proteins. Ligand-bound mGluR2 activates heterotrimeric Gαβγ proteins by exchanging GDP (bound to the Gα) for GTP, which leads to dissociation of GTP-bound Gα from Gβγ. The Gαi subunit inhibits adenylyl cyclase, and thereby decreases intracellular cAMP. The Gβγ subunits, after dissociation from Gαi subunits, also act as a signaling molecule to induce Erk phosphorylation^18^. To know whether glutamate-induced Erk phosphorylation is mediated by Gα and /or Gβγ, we examined the effect of NF023 (an inhibitor for Gαi) and gallein (an inhibitor for Gβγ) on glutamate-induced Erk phosphorylation in primary cultured SC. We found that the glutamate induced increase of Erk phosphorylation was significantly suppressed by gallein to the control level, while NF023 showed no significant effect (Fig. 5A). Furthermore, we found that gallein inhibited glutamate-induced SC proliferation (Fig. 5D). These results suggest that Gβγ plays a role in signaling from mGluR2 to Erk. To demonstrate the role of Gβγ directly, we examined the levels of Erk phosphorylation in SC overexpressing Gβγ proteins. Among variants of Gβγ proteins expressed in SC, we chose to express a well-characterized combination of Gβ1 and Gγ2^18^. We found that SC overexpressing Gβ1 and Gγ2 induced Erk phosphorylation, and also enhanced Nrg1-induced Erk phosphorylation (Fig. 5B). We also found that SC overexpressing Gβ1 and Gγ2 showed enhanced proliferation in response to glutamate (Fig. 5E). These results confirmed the involvement of Gβγ in mGluR2-mediated SC signaling.

Previous reports have shown that intracellular signaling molecules downstream of Gβγ include Src and PI3K. To uncover the involvement of Src and PI3K in glutamate-elicited signaling in SC, we examined glutamate-induced Erk phosphorylation in the presence of Src and PI3K inhibitors^19^,^20^,^21^. We found that Src I, a Src inhibitor, significantly decreased the induction of Erk phosphorylation by glutamate applied with Nrg1. Whereas, inhibition by LY294002, demonstrated no inhibition of Erk phosphorylation (Fig. 5C). These results suggest that Gβγ proteins transmit signaling in SC mainly via Src.

### mGluR2 signaling regulates SC differentiation/myelination

**Figure 6:**
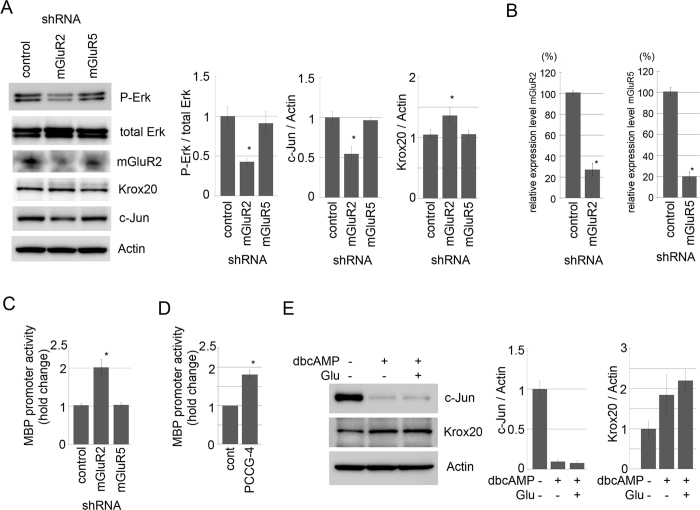
Down-regulation of mGluR2 mediated signaling promotes myelinating SC phenotype. (**A,B**) Representative images of immunoblot analysis (left) and quantified expression levels (right) of phosphorylated Erk, Krox20 and c-Jun expression in SC with RNAi-mediated down-regulation of mGluR2 or mGluR5. β-actin served as a loading control. The quantified expression level was normalized to the total Erk expression level for phosphor Erk, or normalized to the β-actin expression level for Krox20 and c-Jun, relative to the control(Mean ± SD, n = 3). Note that RNAi-mediated mGluR2 down-regulation reduces phosphorylated Erk and c-Jun expression while it induces Krox20 expression in primary cultured SC. Efficiency of RNAi-mediated down-regulation of mGluR2 and mGluR5 expression performed in (**A**) is shown in (**B**) (n = 3, mean expression levels relative to control ± SD). The asterisks indicate significant difference (Student’s t test, *P < 0.05). (**C,D)** Luciferase reporter assay showing that MBP expression is induced by RNAi-mediated down-regulation of mGluR2 (but not by mGluR5) (**C**) or by application of PCCG-4, an mGluR2 antagonist (**D**) (n = 3, Mean ± SD). Asterisks indicate significant difference from control (Student’s t test, *p < 0.05). (**E**) Representative images of immunoblot and quantified expression levels of c-Jun and Krox20 in primary cultured SC after treatment with dbcAMP (1 mM) together with glutamate for 24 h. The quantified expression levels of Krox20 and c-Jun normalized to the β-actin expression level relative to the control (no glutamate/no dbcAMP) are shown. (mean ± SD, n = 3).

In SC, promotion of the differentiated phenotype is strongly associated with inhibition of the proliferation/de-differentiation phenotype; and the same is true for inhibition of differentiation and promotion of proliferation/de-differentiation^7^,^8^,^10^,^22^. Here we have shown that mGluR2 signaling promotes proliferation by enhancing Erk phosphorylation in SC. To examine whether the transcription of genes that designate SC differentiation/de-differentiation phenotypes are regulated together with the induction of Erk phosphorylation, we examined the effect of mGluR2 signaling on SC differentiation. For this purpose, we examined expression of Krox20, a key transcription factor promoting the myelinating phenotype^23^, and c-Jun, a negative regulator of myelination, under RNAi-mediated down-regulation of mGluR2 expression in primary cultured SC. We found increased expression of Krox20 and decreased expression of c-Jun, together with decreased Erk phosphorylation (Fig. 6A). To examine the contribution of mGluR2-elicited possible cross-talk with previously identified SC differentiation/de-differentiation signaling, we examined the effect of mGluR2 signaling on cAMP-induced SC signaling (Fig. 6E). We found that dibutyl-cAMP-induced down-regulation of c-Jun and induction of Krox20 in primary cultured SC are not affected by additional stimulation by glutamate. These results suggest that cAMP signaling in SC is not affected by mGluR2-elicited signaling.

To examine the phenotypic consequence of the Krox20 increase, we performed luciferase reporter assay using SC with mGluR2 down-regulation, to evaluate the transcription status of myelin basic protein (MBP), whose expression is critically important for the myelinating SC phenotype. We found increased transcription of MBP in SC with mGluR2 down-regulation (Fig. 6B,C). Furthermore, we found the similar increase of MBP promotor activity by applying PCCG-4 to primary cultured SC (Fig. 6D). These results suggest that mGluR2 signaling not only stimulate proliferations of SC, but also regulates their differentiation status.

**Figure 7:**
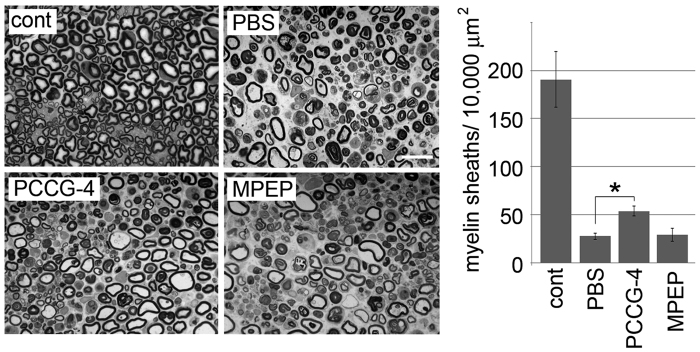
Local administration of mGluR2/3 antagonist inhibits demyelination. Representative photomicrographs of injured sciatic nerve (4 d after transection) cross sections treated locally using Gelfoam with antagonists against indicated mGluRs. Scale bar = 20 μm. For quantification, the number of intact myelin in the indicated area were counted in five independent experiments for each condition (Mean ± SD). Asterisks indicate significant difference from control (Student’s t test, *p < 0.05).

**Figure 8:**
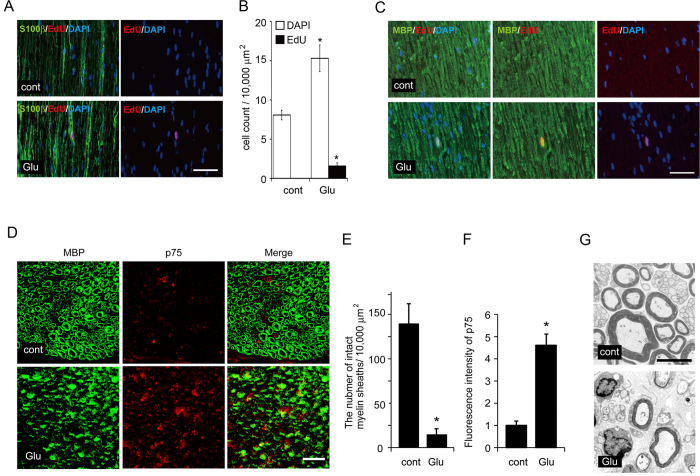
Local administration of glutamate promotes Schwann cell demyelination and proliferation *in vivo*. (**A–C**) Representative photomicrographs of proliferating SC in sciatic nerves locally administered with glutamate for 3d. EdU-incorporated cells were co-stained with S100β (a SC marker) (**A**) or MBP (a myelinating SC marker) (**B**) together with DAPI. Scale bar = 50 μm. The number of EdU-incorporated proliferative SC, and total SC numbers were counted in five independent experiments in each condition (Mean ± SD). Statistical analysis was performed by Student’s t-test, Asterisks indicate significant difference (*p < 0.05). (**D–F**) Local administration of glutamate promotes myelin sheath breakdown and upregulation of p75 expression in SC *in vivo*. (**D**) Representative photomicrographs of transverse sections of sciatic nerves locally administered with glutamate for 2d. Sections were co-stained with p75 (a promyelinating SC marker) and MBP (a myelinating SC marker). Scale bar = 25 μm. (**E**) The quantification of intact myelin sheaths onsections (**E**) and theintensity of p75 immunoreactivity relative to the intensity at Glu(−) condition (**F**) are shown. (n = 3; mean ± SD) The asterisk indicates significant difference (Student’s t test; *p < 0.05). Note that myelin sheath breakdown and upregulation of p75 expression was observed after local administration of glutamate in sciatic nerves. (**G**) Representative photomicrographs for electron microscopic analysis of cross sections of the sciatic nerves locally administered with glutamate for 2d. Scale bar = 5 μm.

Despite the relatively weak induction of the myelinating phenotype by mGluR2 down-regulation in the experimental promoter assay context, we have observed clear promotion of myelination in an *in vitro* myelination assay using an mGluR2/3 antagonist and mGluR2 down-regulation (Figs 1 and 4). To clarify the biological significance of mGluR2 signaling in SC by performing phenotypic analysis under more physiologically relevant situations, we applied PCCG-4 to the area surrounding transected sciatic nerves *in vivo*. We found that direct PCCG-4 application around injured nerves using Gelfoam prevented injury-induced demyelination (Fig. 7). These results support the significance of mGluR2 signaling to inhibit SC differentiation/myelination. To show the proliferation/de-differentiation-promoting effect of mGluR2 signaling in SC in a physiological setting, we examined the effect of applying glutamate directly to the area surrounding sciatic nerves *in vivo*. We found that proliferating SC appeared only when glutamate is applied to the sciatic nerves (Fig. 8A,C). We also found that, in the glutamate-stimulated sciatic nerves, as MBP-expressing SC number decreases EdU-positive proliferating cells and p75-positive de-differentiating cells appear in MBP-expressing SC, indicating that myelinating SC are transformed to proliferating SC in response to glutamate treatment (Fig. 8D). The effect of glutamate on myelinating SC was also confirmed by electron microscopic analysis, in which phagocytosed myelin and non-myelinating SC appeared by glutamate treatment. Together, these results suggest that glutamate serves as a physiological modulator of SC differentiation/proliferation via mGluR2.

## Discussion

Glutamate is an amino acid necessary for protein metabolism in all cells. In the central nervous system (CNS), it is also important as an excitatory neurotransmitter. In the CNS, astrocytes which express GS serve to terminate glutamatergic transsynaptic signaling by uptaking glutamate released from synaptic terminals^24^. In the PNS, on the other hand, while GS is known to be expressed by SC^25^, glutamate is not a neurotransmitter, and the role of GS and glutamate in PNS has remained unclear. Here we established its role as an extracellularly released signaling molecule regulating proliferation and differentiation of SC. Previously identified extracellular signals that regulate SC differentiation status include molecules associated with axonal contact, i.e., cell surface molecules such as NCAM, extracellular matrix molecules such as integrins, and trophic factors such as Nrg1^26^,^27^,^28^. The effect of glutamate on SC is similar to the effect of Nrg1 in that both induce SC proliferation and de-differentiation. We also showed that glutamate-induced potentiation of Nrg1 signaling does not influence cAMP-induced initiation of differentiation, which recapitulates previously reported effect of Nrg1 on glutamate-treated SC^29^. However, glutamate is unique in that it can promote only some of the SC de-differentiation phenotype by promoting phosphorylation of specific tyrosine residues of ErbB2 to activate only a part of its downstream signaling^7^,^8^.

The ErbB2/3 receptor complex can transmit multiple signaling in SC. ErbB2/3 on SC usually takes Nrg1 as a ligand and can associate with a variety of molecules in the SC cytoplasm, which transmit different signals leading to multiple phenotypic consequences^30^. It was previously reported that membrane-bound Nrg1 can regulate myelination, while secretory forms of Nrg1 is inhibitory to myelination^5^,^7^,^31^. Another report showed that soluble Nrg1 has multiple functions in a manner dependent on its concentration. Nrg1 is myelination-promotive in the lower concentration range, while it is inhibitory to myelination at higher concentrations^32^. Additional complexity can come from subcellular signaling downstream of ErbB2/3. We found that Y1221/1222 is preferentially phosphorylated in the ErbB2 molecule by activation of the mGluR2 signal. Y1221/1222 phosphorylation is known to constitute a docking site for cytoplasmic proteins of the ErbB2 signal transduction cascade, while Y877 regulates ErbB2 kinase activity^33^. The glutamate-induced Y1221/1222 phophorylation of ErbB2 may be necessary to determine the ErbB2 signaling direction, which leads to enhanced Erk, but not Akt phosphorylation in SC. Pro-myelinating SC during development or in degenerating/regenerating nerves first need to proliferate before contacting axons to make proper alignment with them. As we reported previously, glutamate is produced and secreted by pro-myelinating SC^15^. We presume that glutamate promotes the non-myelinating phenotype of SC and enhance their proliferation by limiting the function of Nrg1/ErbB2 signaling, before SC come in contact with, or proximity of axons to be stimulated by other signals. These other signals include membrane-bound Nrg1, as well as integrins and extracellular matrix molecules, that cause migration, alignment, and myelination.

We show here that glutamate application on normal sciatic nerve can induce de-differentiation of myelinating SC to initiate proliferation. We have shown previously that forced expression of GS in primary cultured SC promotes myelination *in vitro*^15^. These effects of mGluR2-mediated signaling on SC differentiation seem very strong and important, almost comparable to application of Nrg1 or expression of Krox-20, which are the master regulators of SC phenotype^23^. This strong effect is in contrast to the subtle induction of Erk phosphorylation observed by glutamate application or weak promotion of myelinating phenotype by mGluR2 down-regulation in SC. One possible explanation for this difference is that mGluR2 signaling exerts strong phenotypic consequences by promoting de-differentiation, while inhibiting differentiation at the same time. Indeed, we observed expression of p75 and/or initiation of proliferation in cells maintaining expression of MBP in response to glutamate, and that mGluR2 down-regulation induce expression of krox20, an inducer of SC myelination. The combinatorial effect could be very strong *in vivo* settings compared with apparent induction capability of Erk phosphorylation *in vitro*. Another possibility is that Nrg1 is continuously produced and always present at low level in physiological peripheral nerves, and so therefore glutamate can always exert strong effects *in vivo* despite its effect on cultured SC *in vitro*.

We showed here that inhibition of mGluR2 signaling in SC not only promotes myelination, but also prevents demyelination. This effect may be relevant for therapeutic application of the mGluR2 inhibition against demyelinating disorders in PNS, such as Charcot Marie Tooth disease type 1 (CMT1). CMT is one of the most common genetic disorders, affecting one out of every 2500 people, and causes severe motor and sensory deficits resulting in significant patient morbidity and mortality, but no treatment against CMT exists thus far^34^. De-myelination in CMT caused by mutations in major myelin protein genes such as P0^35^,^36^ and PMP22^37^ is typically characterized by repeating cycles of demyelination and incomplete myelination, so preventing de-myelination is critically important for treatment of CMT1. Since a number of compounds for modulating mGluR function have already been accumulated to study neurotransmission in CNS^38^,^39^, some of these may prove to be as useful tools to develop treatment for CMT1 in the near future.

## Materials and Methods

### Animals and surgical procedures

The experimental procedures performed on mice and rats were carried out in accordance with the approved guidelines from the Ministry of Health, Labor and Welfare of Japan. The experimental protocol was approved by the Animal Welfare Committee in the National Center of Neurology and Psychiatry.

Sciatic nerves of eight- to eleven-week old mice were treated with glutamate or PBS using collagen-gel matrices, Gelfoam (Pfizer) for 3 days. Sciatic nerve transection experiments were performed as previously described^40^. The mice were then injected with EdU (Invitrogen) for 2 hours, as described in the manufacturer’s protocol, before perfusion with fixative for histological analysis.

For analysis of *in vivo* effects of reagents, mGluR2/3 antagonist (2S,1′S,2′S,3′R)-2-(2′-carboxy-3′-phenylcyclopropyl)glycine (PCCG-4, 100 μM; Enzo Life Sci.) and mGluR5 antagonists 6-Methyl-2-(phenylethynyl)pyridine (MPEP, 10 μM; Sigma) were administered locally using Gelfoam around sciatic nerves of 8 to 11 week old mice for 4 days prior to collection for histological analysis.

### Expression plasmids and viral vectors

His-tagged Gβ1 (His-Gb1) and His-Gγ2 expression vectors were generated by amplifying the full-length coding region of the Gβ1 cDNA (GenBank accession number NM_030987) and Gγ2 (GenBank accession number NM_031754); and then cloned into pcDNA4-His MAX at the EcoR I site. The integrity of the clones was verified by nucleotide sequence analysis. Luciferase reporter plasmid MBP-luc was a gift from Dr. Lawrence Wrabetz. CMV-Renilla.luciferase was purchased from Promega.

For cosmid vector construction, His-tagged Gβ1 and Gγ2 were cloned into the pAxCALNLwtit2 cosmid vector (Takara Bio). Recombinant adenovirus was generated using the Takara adenovirus expression kit following the manufacturer’s instructions. The purification and concentration of adenoviral vectors were carried out by cesium chloride density gradient centrifugation and Centricon Centrifugal Filter Devices (Millipore). Viral titres were determined with a plaque-forming assay in HEK293 cells as previously described^41^. For experiments using lentiviral vectors, control and mGluR2-specific short hairpin RNAs in the pLKO.1 puromycin-resistant vector were purchased from Sigma. The following clones were used: mGluR 2 (MISSION shRNA: TRCN0000219563 ), mGluR 3 (MISSION shRNA: TRCN000025767), mGluR 5 (MISSION shRNA: TRCN0000219566), and non-target control (MISSION shRNA: SHC002). Lentiviral packaging was performed using HEK293T cells as previously described^42^. The efficiency of infection to SC (GFP-positive cells/total cells) routinely reached >95%.

### Primary SC culture, transfection, and luciferase reporter assay

Primary SC cultures were prepared as described previously^15^.

mGluR antagonists were applied to the cultures at following concentrations: mGluR1 antagonist (RS)-1-aminoindan-1,5-dicarboxylic acid (AIDA, 100 μM; Tocris biosci.), mGluR2/3 antagonists PCCG-4 (100 μM) and mGluR5 antagonist MPEP (10 μM). In some experiments, SC were treated with NF023, a Gαi inhibitor, at 10 μM, Gβγ Modulator II, Gallein (Millipore) at 400 nM, dibutyryl cyclic AMP (dbcAMP) at 1 mM, ErbB2 inhibitor PKI166 (a gift from Novartis) at 200 nM.

For analyzing Erk phosphorylation status, SC were serum-starved with 1% FBS DMEM for 6 hours, followed by 1% FBS DMEM containing Nrg1 and indicated reagents for 30 min.

For proliferation analysis, SC were treated with 2 mM glutamate, together with indicated mGluR antagonists for 24 h later. EdU (20 μM) was added 2 h before fixation for detection by Click-iT EdU Imaging Kit (Invitrogen). Quantification of proliferating cell number was performed by counting the number of Edu-labeled cells under microscope in four randomly selected fields per each condition using 5x objective lens.

For reporter assays, the SC were transfected with MBP- firefly luciferase and CMV- Renilla luciferase using the NEPA21 electroporator (NEPA GENE, Japan). After transfection, Schwann cells were cultured for 3 days before harvesting for luciferase activity measurement using dual luciferase assay kit (Promega).

### Immunoblot

Cells were washed and lysed with lysis buffer containing 50 mM Tris-HCl, pH8.0, 150 mM NaCl, 1% NP-40, 0.5% DOC, 0.1% SDS. The lysate was subject to SDS-PAGE and immunoblot analysis with a standard procedure^43^ using antibodies against Erk (Cell signaling, Danvers, MA), phospho-Erk (Cell signaling) His (MBL), Krox20 (BioLegend), ErbB2(NeoMarkers), pY1221/1222-ErbB2(Abcam), pY877-ErbB2(Abcam), and pY1248-ErbB2(Abcam).

### Reverse transcription-PCR

Total RNA was isolated from indicated cells using RNeasy MiniKit (Qiagen), and first-strand cDNA was obtained with SuperScript III, according to the manufacturer’s protocol (Invitrogen). PCR was performed using LA Taq (Takara Bio) with the following primer sets: mGluR1, 5′-GGAATGGTACGATCTGTGTG-3′, 5′-CCACTCAAGATAACGGACAG-3′; mGluR2, 5′-CACCTGCATCATCTGGCTGGC-3′, 5′-GGGATCCAGACCCTTGAC-3′; mGluR3, 5′-CCTCAACAGGTTCAGCGTCAG-3′, 5′-TGTGAGCACTTTGTCTAACAG-3′; mGluR4, 5′-ACAGTCAGCCGACAAGCTGTACAT-3′, 5′-ATGGTTGGTGTAGGTGACGTAGGT-3′; mGluR5, 5′-GATCCAGCAGCCTAGTCAAC-3′, 5′-GTCGCTGCCACAAATGTTGC-3′; mGluR6, 5′-GAGGTGTGCCTGAGACCTTC-3′, 5′-GCTCAGTGACACAGTTAGTG-3′; mGluR7, 5′-CATGTCATCAAGGCTGTCAC-3′, 5′-GGAACAGGTTAGATAACCAG-3′; mGluR8, 5′-CAATGTACACCACGTGCATC-3′, 5′-CATTCCCAGAGACACTGAAG-3′. GAPDH, 5′-ACAAAATGGTGAAGGTCGGTGTGA-3′, 5′-AGCTTCCCATTCTCGGCCTTGAC-3′; S100b, 5′-TGGTTGCCCTCATTGATGTCT-3′ 5′-CCCATCCCCATCTTCGTCC-3′; Brn3a, 5′-CAGGAGTCCCATGTAAGA-3′, 5′-ACAGGGAAACACTTCTGC-3′.

Real time quantitative PCR (qRT-PCR) was performed on the ABI Prism 7300 System (Applied Biosystems) as previously described^15^. The fluorescence data were quantitatively analyzed using serial dilutions of the control samples included in each reaction to produce a standard curve.

### *In vitro* myelination assay

*In vitro* myelination assays were performed as described previously^15^. In some experiments, myelination was induced with mGluRs antagonist at concentrations mentioned above. For analysis of myelin profiles by immunocytochemistry, 10 days after initiation of myelination, the culture was fixed with 4% paraformaldehyde/PBS for 5 min. Myelination profiles were visualized by immunocytochemical detection of myelin basic protein (MBP) using anti-MBP antibody (Covance) followed by Alexa-594-conjugated anti-mouse IgG (Invitrogen). For quantification, myelination profiles in 5 randomly chosen fields using a 20x objective lens were counted and the number of myelinated nerve fibers per arbitrary unit area was calculated. Statistical analysis was performed by Student’s *t* test. All data are reported as mean ± SD; *p* value of less than 0.05 is considered significant.

### Histological analysis

Immunohistochemistry was performed as previously described^15^,^44^. For cell proliferation assay, cryostat sections of mouse sciatic nerve tissues were subject to detection using Alexa-594-conjugated EdU detection kit (Invitrogen). The sections were co-immunostained for S100β (Sigma) or MBP using Alexa-488-conjugated anti-mouse antibody (Invitrogen) for visualization. For quantitative analysis of proliferating cells, the number of EdU-positive cells was calculated and normalized to total cell numbers per arbitrary unit area. Statistical analysis was performed by Student’s *t* test. All data are reported as mean ± SD; *p* value of less than 0.05 is considered significant.

Toluidine blue staining of nerve samples was performed as previously described^40^.

For electron microscopic analysis, mice were perfusion fixed transcardially with 2% paraformaldehyde and 2.5% glutaraldehyde in PBS. Fixed sciatic nerves were removed and embedded in 3% agarose in PBS. Transverse sections (150 um thickness) were prepared by using a Microslicer (DTK-3000; Dosaka), and the sections of the location 3 mm distal to the transected site were selected under microscopic observation. The selected sections were rinsed, osmicated, dehydrated, and embedded in epoxy resin. Ultrathin sections were prepared, stained with lead citrate and uranyl acetate, and observed under a transmission electron microscope (Tecnai Spirit, FEI).

## Additional Information

**How to cite this article**: Saitoh, F. *et al*. Glutamate signals through mGluR2 to control Schwann cell differentiation and proliferation. *Sci. Rep*. **6**, 29856; doi: 10.1038/srep29856 (2016).
